# The Deoxynucleoside Triphosphate Triphosphohydrolase Activity of SAMHD1 Protein Contributes to the Mitochondrial DNA Depletion Associated with Genetic Deficiency of Deoxyguanosine Kinase*

**DOI:** 10.1074/jbc.M115.675082

**Published:** 2015-09-04

**Authors:** Elisa Franzolin, Cristiano Salata, Vera Bianchi, Chiara Rampazzo

**Affiliations:** From the ‡ Department of Biology, University of Padova, 35131 Padova, Italy and; § Department of Molecular Medicine, University of Padova, 35121 Padova, Italy

**Keywords:** gene silencing, mitochondrial disease, mitochondrial DNA (mtDNA), nucleoside/nucleotide metabolism, phosphatase, ribonucleotide reductase, dGTP pool, mitochondrial deoxynucleoside salvage, mtDNA precursors

## Abstract

The dNTP triphosphohydrolase SAMHD1 is a nuclear antiviral host restriction factor limiting HIV-1 infection in macrophages and a major regulator of dNTP concentrations in human cells. In normal human fibroblasts its expression increases during quiescence, contributing to the small dNTP pool sizes of these cells. Down-regulation of SAMHD1 by siRNA expands all four dNTP pools, with dGTP undergoing the largest relative increase. The deoxyguanosine released by SAMHD1 from dGTP can be phosphorylated inside mitochondria by deoxyguanosine kinase (dGK) or degraded in the cytosol by purine nucleoside phosphorylase. Genetic mutations of dGK cause mitochondrial (mt) DNA depletion in noncycling cells and hepato-cerebral mtDNA depletion syndrome in humans. We studied if SAMHD1 and dGK interact in the regulation of the dGTP pool during quiescence employing dGK-mutated skin fibroblasts derived from three unrelated patients. In the presence of SAMHD1 quiescent mutant fibroblasts manifested mt dNTP pool imbalance and mtDNA depletion. When SAMHD1 was silenced by siRNA transfection the composition of the mt dNTP pool approached that of the controls, and mtDNA copy number increased, compensating the depletion to various degrees in the different mutant fibroblasts. Chemical inhibition of purine nucleoside phosphorylase did not improve deoxyguanosine recycling by dGK in WT cells. We conclude that the activity of SAMHD1 contributes to the pathological phenotype of dGK deficiency. Our results prove the importance of SAMHD1 in the regulation of all dNTP pools and suggest that dGK inside mitochondria has the function of recycling the deoxyguanosine derived from endogenous dGTP degraded by SAMHD1 in the nucleus.

## Teaser

**Background:** Deoxyguanosine kinase (dGK) deficiency causes mtDNA depletion in quiescent cells where SAMHD1 restricts dGTP concentration.

**Results:** siRNA silencing of SAMHD1 corrects mt dNTP imbalance boosting mtDNA content in quiescent dGK-mutated human fibroblasts.

**Conclusion:** In noncycling cells SAMHD1 activity worsens the phenotype of dGK genetic deficiency, limiting dGTP supply.

**Significance:** Nuclear SAMHD1 and mitochondrial dGK functionally interact in the regulation of mt dGTP for mtDNA maintenance.

## Introduction

In mammalian cells the concentrations of deoxyribonucleoside triphosphates (dNTPs) are regulated by an interplay of synthesis and degradation (1). Synthesis of dNTPs occurs through two pathways, *de novo* synthesis of dNDPs in the cytosol by ribonucleotide reductase (RNR)^3^ followed by phosphorylation to dNTPs by nucleoside diphosphate kinase and salvage of deoxyribonucleosides by two parallel sets of deoxynucleoside and -nucleotide kinases in the cytosol and in mitochondria (2). The rate-limiting step of the salvage pathway is the phosphorylation of deoxyribonucleosides to their monophosphates, catalyzed by thymidine kinase 1 (TK1) and deoxycytidine kinase (dCK) outside mitochondria and thymidine kinase 2 (TK2) and deoxyguanosine kinase (dGK) inside mitochondria. The substrate specificity of each pair of enzymes permits the phosphorylation of all physiological deoxyribonucleosides in both instances (3). Degradation of deoxyribonucleotides is carried out by 5′-nucleotidases that dephosphorylate nucleoside monophosphates to deoxyribonucleosides, inverting the kinase reactions. Deoxyribonucleoside kinases and 5′-nucleotidases generate substrate cycles that regulate the exchange of deoxyribonucleosides across the plasma membrane, thus modulating the overall dNTP pool size (4). Intracellular deoxyribonucleosides are also degraded by phosphorylases and deaminases that actively participate in the regulation of dNTP pools, as demonstrated by the large pool imbalances and severe pathologies arising from their genetic deficiency (5, 6). The catabolic enzymes mentioned so far are constitutively expressed but show tissue-specific variations.

The dNTP triphosphohydrolase SAMHD1 is a new addition to the set of catabolic enzymes and has recently emerged as a main actor in the regulation of dNTP metabolism (7–10). The enzyme cleaves dNTPs to deoxyribonucleosides and triphosphate, reversing in a single step the entire salvage pathway, which may interfere with the *de novo* pathway as dNTPs are allosteric effectors of ribonucleotide reductase. The impact of SAMHD1 activity on dNTP pools is surprisingly strong. Down-regulation of the enzyme by siRNA silencing, ubiquitin-dependent proteolysis, or genetic inactivation increases the concentrations of all dNTPs (9–13) in human fibroblasts, especially that of dGTP (10). This effect is particularly marked in quiescent or differentiated cells where the level of SAMHD1 is higher than during proliferation.

The combination of synthetic and catabolic reactions provides a fine cyclic regulation of dNTP pools in tune with nuclear DNA replication. Pool sizes expand at the onset of S-phase when the request for dNTPs suddenly increases and diminish when DNA replication is completed and ribonucleotide reduction is turned off by degradation of the RNR small subunit R2. In the absence of SAMHD1 the cyclic variation of dNTP pools is lost, and the pools remain high also when the frequency of S-phase cells is low (10). In normal human fibroblasts silenced for SAMHD1 the accumulation of dNTPs outside S-phase interferes with the normal progression of the cell cycle, delaying cells in G_1_ (10).

SAMHD1 is a modular protein. The protein sequence contains an N-terminal nuclear localization signal followed by a sterile α-motif domain and a catalytic HD-domain typical of a large family of phosphohydrolases. A short C-terminal stretch is important for interactions with proteins regulating SAMHD1 stability (14) and contains a conserved threonine residue (Thr-592) phosphorylated in proliferating cells by cyclin A-dependent cyclin-dependent kinases (15–18). The effects of Thr-592 phosphorylation on SAMHD1 function are incompletely understood. Crystallographic studies of the isolated HD domain demonstrate that SAMHD1 is a homotetrameric enzyme allosterically regulated by dNTPs, similar to RNR (19–22). A SAMHD1 tetramer contains four catalytic sites specific for deoxyribonucleoside triphosphates and eight allosteric sites, four specific for dGTP or GTP and four binding all four dNTPs, with the lowest affinity for dCTP (23). The solved structures of SAMHD1 complexed with effectors and substrates illustrate the structural basis for both allosteric control and substrate specificity (19–22). Exonuclease activity has also been reported for SAMHD1 (24), but the nature of the preferred substrate is controversial. A recent study (25) showed that the triphosphohydrolase and nuclease activities of SAMHD1 are inactivated independently by genetic mutations occurring *in vivo* in patients affected by Aicardi Goutières syndrome (26). So far, nobody has shown how the solved three-dimensional structure of active SAMHD1 can accommodate both enzyme activities.

**FIGURE 1.**
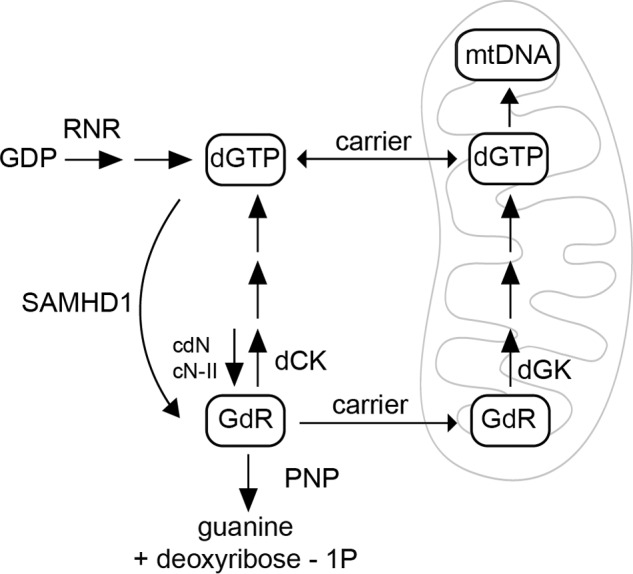
**Interaction between SAMHD1 and deoxyguanosine kinase activities in the regulation of mitochondrial dGTP.** Synthesis of dGTP occurs in the cytosol by *de novo* synthesis catalyzed by RNR and by salvage of GdR catalyzed by dCK. The two synthetic pathways are counteracted by catabolic activities distributed between the nucleus and cytoplasm. SAMHD1 degrades dGTP to GdR in the nucleus, whereas in the cytosol 5′-nucleotidases (cdN and cN-II) dephosphorylate dGMP to GdR, and PNP degrades GdR to free guanine. Due to the permeability of the nuclear envelope to nucleotides and nucleosides, nuclear and cytosolic pools represent a single kinetic compartment. The mitochondrial dGTP pool derives most of its components from the cytosol via nucleotide and nucleoside carriers in the inner mt membrane. In mitochondria, a separate salvage pathway, depending on dGK, recycles GdR, creating a dGTP pool protected from the catabolic activity of SAMHD1.

Although DNA synthesis requires roughly equimolar amounts of the four dNTPs, these occur at unequal concentrations in cells (27, 28). Pyrimidine dNTPs are generally more abundant than purine dNTPs, with dGTP as the smallest pool. The advantages of small dGTP pools may be rationalized. High dGTP concentrations are toxic because dGTP stimulates the reduction of ADP by RNR, and dATP is the main negative effector of the enzyme (2). Moreover, dGTP is highly mutagenic (29), is prone to oxidative damage, and in the oxidized form can be incorporated into DNA, reducing DNA polymerase γ replication fidelity with destabilizing consequences (30, 31). An exception to the advantages of small dGTP pools is provided by rat tissue mitochondria, reported to contain huge amounts of dGTP (32). The control of dGTP concentration so far has been considered to result from a balance between the efficiency of dGTP *de novo* synthesis and the catabolic action of 5′-nucleotidases cdN and cN-II, the two main intracellular 5′-nucleotidases degrading dGMP (4), and of purine nucleoside phosphorylase (PNP) that degrades the deoxyguanosine produced by the nucleotidases (Fig. 1). The large effects of SAMHD1 ablation on the concentration of dGTP in various types of mammalian cells show that an important piece of the puzzle was missing. SAMHD1 limits the size of the dGTP pool, especially in quiescent cells, which in culture seem to produce more dGTP than they need.

Cells contain two separate dGTP pools, a very small mitochondrial pool enclosed within the mt inner membrane, and a 10-fold larger extramitochondrial pool distributed between the cytosol and nucleus. The two pools communicate through nucleotide (33, 34) and nucleoside (35, 36) carriers in the mt membrane such that guanine deoxynucleotides produced *de novo* in the cytosol are the main source of mt dGTP both during proliferation and quiescence (37). However, the presence of a mitochondrial salvage pathway that converts deoxyguanosine (GdR) to dGTP suggests that the import of dGTP from the cytoplasm does not suffice for mtDNA synthesis. Indeed, genetic deficiency of dGK causes a mtDNA depletion syndrome with a specific hepato-cerebral phenotype (38). As is the case with other forms of mtDNA depletion syndrome, the enzyme deficiency targets differentiated cells that have small dNTP pools. However, the tissue-specific effects typical of dGK deficiency remain unexplained. The mtDNA depletion in dGK-mutated individuals is commonly attributed to an insufficient supply of dGTP, an explanation based on limited direct experimental evidence published before the discovery of SAMHD1 (39). Wishing to understand how SAMHD1 interacts with other enzymes in the regulation of dNTP pools, we set out to investigate its impact on the mt pools of cells with defective mt salvage of purine deoxyribonucleosides. We found that dGK deficiency reduces mt dGTP in human fibroblasts only moderately but causes mt dNTP imbalances and mtDNA depletion during quiescence. SAMHD1 down-regulation by siRNA transfection restores mt pool balance and boosts mtDNA copy number, compensating the mtDNA depletion to various degrees in different mutant lines. Interestingly, the activity of SAMHD1 in the nucleus affects the mt dNTP pool by reducing the cellular dGTP content. We propose that inside mitochondria dGK is required to recycle the GdR released from extramitochondrial dGTP by SAMHD1.

## Experimental Procedures

### 

#### 

##### Materials, Cell Lines, and Cell Growth

[5-^3^H]Deoxycytidine (9000 cpm/pmol) and [8-^3^H]deoxyguanosine (6000 cpm/pmol) were from Moravek (Brea, CA). Immucillin H was a gift of Dr. Vern Schramm (Yeshiva University, New York).

We used three lines of skin fibroblasts derived from patients with inactivating mutations in *DGUOK*, the gene for deoxyguanosine kinase. Patient 1 carried the heterozygous mutations D255X and E165V (40), patient 2 (*i.e.* patient 14 in Table 3 of Ref. 41) was heterozygous for two different mutations, *i.e.* K201fs214X; IVS4 splicing site, and patient 3 (42) carried the homozygous mutation L250S. Patient 1 fibroblasts were donated by Dr R. Martì (Hospital Vall d'Hebron, Barcelona, Spain). Fibroblasts from patients 2 and 3 were donated by Dr Massimo Zeviani from the collection of the Neurological Institute C. Besta, Milano (Italy). Three lines of age-matched control skin fibroblasts were available in our laboratory. All fibroblasts were grown in DMEM with 4.5 g of glucose per liter, 10% (v/v) fetal calf serum, nonessential amino acids, and antibiotics.

##### Transfections with siRNAs, RNA Extraction, Reverse Transcription, and Real-time PCR

All procedures were performed as described in Franzolin *et al.* (10). The siRNAs used were from Qiagen: AllStar negative control siRNA (catalog no.1027281), siRNA 3 as in Laguette *et al.* (43), siRNA 4 (catalog no. SI00710500), and siRNA 7 (catalog no. SI04243673).

##### Western Blotting

Samples of 1–2 million cells were collected by centrifugation, washed with PBS, and lysed with radioimmunoprecipitation assay buffer (10 mm Tris·HCl, pH 7.4, 100 mm NaCl, 1% sodium deoxycholate, 0.1% SDS, 1% Nonidet P-40) containing a mixture of protease inhibitors for mammalian cells (Sigma). The extracts were centrifuged at 19,000 × *g* for 20 min, the protein concentrations of the supernatant solutions were determined by the BCA protein assay (Pierce), and appropriate amounts of the cleared supernatants were loaded on precast gels, 7.5% (Bio-Rad) for R1 and SAMHD1 separation or Any-Kd gels (Bio-Rad) for R2, p53R2, β actin, and GAPDH, and electrophoresed. The proteins were blotted on Hybond-C extra (GE Healthcare) in the case of ribonucleotide reductase subunits R1, R2, p53R2, β actin, and GAPDH and on PVDF (Millipore) for SAMHD1. Both membranes were saturated with 2% ECL Blocking Agent (GE Healthcare) for 1 h at room temperature and incubated overnight at 4 °C with the following primary antibodies: anti-SAMHD1 1A1 (Abcam), dilution 1:4000; anti-R1 T16 (Santa Cruz) 1:1000; anti-R2 N18 (Santa Cruz) 1:2000; anti-p53R2 N16 (Santa Cruz) 1:1000; anti-GAPDH (Millipore) 1:1000; anti-β actin (Sigma) 1:4000. After 3 washings with PBS + 0.05% Tween 20 for 10 min, the membranes were incubated with horseradish peroxidase-conjugated secondary antibodies (dilution 1:80,000) for 1 h at room temperature. Then the membranes were washed and developed with a chemiluminescence ECL kit (LiteAblotTurbo, Euroclone). The signals were detected on Kodak films and quantified with ImageJ software.

##### Quantitation of dNTP Pools

Cells were washed with ice-cold PBS, scraped from the plates, and homogenized by repeated aspiration through needles of 0.45 × 23 mm. Cytosolic and mt nucleotides were separated by differential centrifugation of cell homogenates, and nucleotide pools were extracted with ice-cold methanol as described (44). The concentrations of the four dNTPs were determined by an enzymatic assay (45) modified as described in Ferraro *et al.* (46). In some experiments, before extracting the nucleotide pools the quiescent cultures were treated for 24 h with 0.5 μm immucillin H to inhibit purine nucleoside phosphorylase (47).

##### Enzyme Assays

Whole cell extracts were prepared as described earlier (48), adding a mixture of mammalian protease inhibitors (Sigma) to the lysis buffer (Tris·HCl, pH 7.5, 10 mm 0.5% Triton X, 2 mm EDTA, 1 mm DTT). Protein concentration was measured by the colorimetric procedure of Bradford with bovine serum albumin as the standard. All enzymatic assays were run with two different aliquots of extract to check for proportionality. Citrate synthase was measured as detailed in Shepherd and Garland (49). Deoxyguanosine and deoxycytidine kinases were tested by incubating the extracts with the radioactive precursor for 60 min in 40 μl of reaction mix and then spotting a 35-μl aliquot on DE 81 paper as described in Leanza *et al.* (37). The specific radioactivity of the labeled substrates was diluted 5–10-fold with non-labeled deoxynucleosides. The activity of dGK was measured with 1 μm [^3^H]deoxyguanosine (1800 cpm/pmol) in the presence of 0.5 mm nonradioactive CdR to inhibit deoxycytidine kinase and 0.5 μm immucillin H to prevent the degradation of deoxyguanosine. The activity of deoxycytidine kinase was determined with 1 μm [^3^H]CdR (1100 cpm/pmol). We express enzyme activities as pmol of product min^−1^ mg^−1^ of protein extract.

##### mtDNA Quantification

We determined mtDNA copy numbers with the TaqMan probe system and Applied Biosystem 7500 real-time PCR as described in Andreu *et al.* (50). Genomic DNA was extracted by Puregene Core kit B (Qiagen). Mitochondrial rRNA 12S TaqMan probe 6FAM-5′-TGCCAGCCACCGCG-3′-MGB (Applied Biosystems) and primers rRNA 12S forward (5′-CCACGGGAAACAGCAGTGATT-3′) and reverse (5′-CTATTGACTTGGGTTAATCGTGTGA-3′) were used to quantify mtDNA. For nuclear DNA, we used RNase P primers and probe VIC mix (Applied Biosystems). To quantify mtDNA and nuclear DNA we used calibration curves generated by serial dilution of a mixture of plasmids carrying the two PCR amplicons. Each DNA sample was analyzed in triplicate.

##### Flow-cytometric Determinations of Cell-cycle Distribution and Mitochondrial Mass

Cell cycle distribution in fibroblast cultures was determined by flow cytometry after propidium iodide staining of fixed resuspended cells, with a BD Biosciences FACSCanto II Flow cytometer. To measure mitochondrial mass 0.8 × 10^6^ cells were detached, washed once, and incubated under rotation in 400 μl of 0.2 μm nonyl acridine orange (Sigma) at 37 °C for 10 min. Then cells were washed once in 10 ml of PBS and centrifuged. The pellet was resuspended in 700 μl of PBS and analyzed immediately by flow cytometry.

##### Statistics

Where appropriate, statistical analyses (two-way analysis of variance with Bonferroni post test) were performed using GraphPad Prism 5 (GraphPad Software, La Jolla, CA).

## Results

The concentration of dGTP in human cells is strongly influenced by the activity of SAMHD1, particularly in noncycling cells that contain higher amounts of the protein than cycling cells. When the enzyme is down-regulated, all four dNTP pools expand, with dGTP undergoing the largest increase (10). The deoxyguanosine released from dGTP by SAMHD1 can be recycled through the salvage pathway via phosphorylation by dGK. To investigate possible functional interactions between the two enzymes, we employed skin fibroblasts derived from three unrelated mtDNA depletion syndrome patients (40–42) with different inactivating mutations in DGUOK, the gene coding for deoxyguanosine kinase, and compared them with WT human fibroblasts.

### 

#### 

##### Characterization of the dGK-mutated Fibroblast Lines

The two kinases that phosphorylate GdR in the cells, dCK and dGK, undergo opposite variations when normal skin fibroblasts pass from proliferation to quiescence, with dCK activity declining and dGK activity increasing (37). We measured the activities of the two enzymes with their preferred substrates, CdR and GdR, respectively, in whole cell extracts from proliferating and quiescent WT and dGK-mutated fibroblasts (Table 1). In the dGK assay the addition of nonradioactive CdR inhibited the phosphorylation of GdR by dCK present in the extracts (37). The activity of dGK was one order of magnitude lower in the mutant fibroblasts than in the WT controls and hardly changed during quiescence, in contrast to that of the controls. The activity of dCK was between 15 and 20 pmol/min/mg protein in all cell lines after 48 h in culture and decreased with time, reaching after 10 days of quiescence values 3-fold lower in the controls and ∼2-fold lower in the mutants.

**TABLE 1:** **Enzyme activities of deoxycytidine kinase and deoxyguanosine kinase in whole cell extracts from proliferating or quiescent control and dGK-mutated skin fibroblasts** Deoxycytidine kinase was tested with 1 μm [5-^3^H]CdR and deoxyguanosine kinase with 1 μm [8-^3^H]GdR. Values are specific activities calculated in duplicate samples from two experiments ± S.E. C, WT controls; P, dGK-mutated patient lines.

Cell line	dCK activity			dGK activity		
	48 h	72 h	Quiescence	48 h	72 h	Quiescence
	*pmol/min/mg*			*pmol/min/mg*		
C1	19.3 ± 0.8	13.6 ± 0.6	7.3 ± 0.1	0.31 ± 0.02	0.31 ± 0.06	0.61 ± 0.18
C2	17.1 ± 0.2	12.9 ± 0.2	5.8 ± 0.2	0.20 ± 0.02	0.41 ± 0.03	0.43 ± 0.02
P1	14.8 ± 0.9	10.7 ± 0.4	9.5 ± 0.3	≤0.01	0.03 ± 0.01	0.02 ± 0.00
P2	14.5 ± 0.2	13.5 ± 0.1	8.1 ± 1.4	0.04 ± 0.01	0.05 ± 0.00	≤0.01
P3	16.5 ± 0.3	11.4 ± 0.2	8.0 ± 1.2	0.04 ± 0.01	0.16 ± 0.01	≤0.01

**FIGURE 2.**
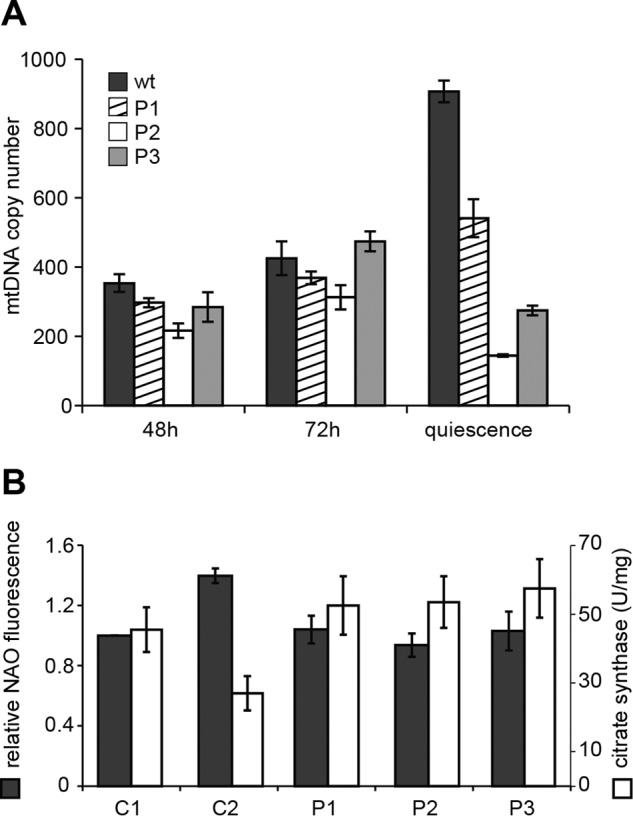
**mtDNA copy number and mitochondrial mass in WT and dGK-mutated fibroblasts.** *A*, we measured mtDNA copy number per nuclear genome in proliferating (48 and 72 h) and quiescent cultures of three lines of wt skin fibroblasts (C1, C2, C3) and three dGK-mutated lines (P1, P2, P3). Control values were combined (wt). All data are the means ± S.E. from at least two experiments for each cell line analyzed in triplicate. *B*, mitochondrial mass was evaluated by measuring citrate synthase activity (units (*U*) = nmol/min) in whole cell extracts and by normalizing the nonyl acridine orange (*NAO*) fluorescence of each line determined by flow cytometry by that of control C1. Data are the means ± S.E. from two experiments.

**FIGURE 3.**
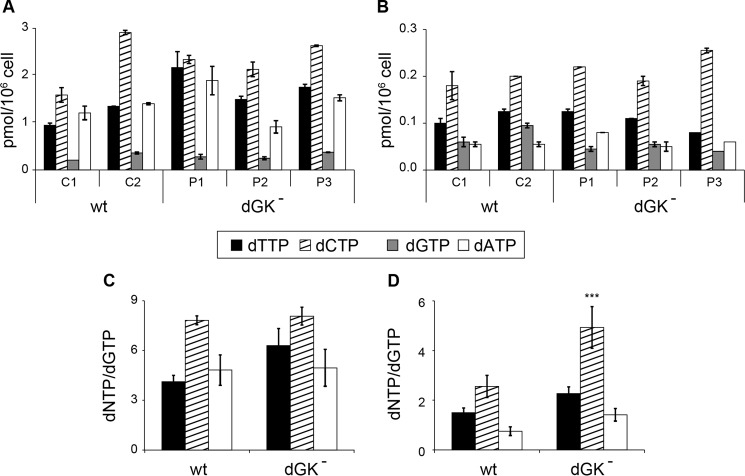
**Cytosolic and mitochondrial dNTP pool sizes in quiescent skin fibroblasts.** Cytosolic (*A*) and mitochondrial (*B*) dNTP pools were measured in quiescent cultures of two WT (*C1*, *C2*) and three dGK-mutated (*P1*, *P2*, *P3*) lines of skin fibroblasts. To compare the two groups we calculated the mean ratios between the size of each dNTP pool and that of dGTP in the cytosol (*C*) and mitochondria (*D*) of WT or dGK-mutated fibroblasts. Data are the means ± S.E. from two experiments for each cell line analyzed in duplicate. *Asterisks* indicate a significant difference (***, *p* < 0.001) of the dCTP/dGTP ratio in mutant fibroblasts relative to controls.

During proliferation the P2 mutant line showed a 30–40% depletion of mtDNA compared with the controls, whereas in the other two mutants the content of mtDNA was similar to that of WT fibroblasts (Fig. 2*A*). After 10 days of quiescence all three mutant lines contained significantly less mtDNA than the WT fibroblasts, in agreement with previous reports of mtDNA depletion in cultured dGK-deficient fibroblasts (39, 51). The low mtDNA content was not accompanied by a reduced number of mitochondria in the mutants. We determined mt mass by flow cytometry in quiescent cells stained with nonyl acridine orange and by measuring the activity of citrate synthase in cell extracts. Both methods showed in mutant cells values of mt mass within the range observed in control fibroblasts (Fig. 2*B*). The depletion of mtDNA during quiescence may depend on an insufficient supply of dGTP in mitochondria. We measured the concentrations of the four dNTPs in the cytosolic and mitochondrial pools of quiescent WT and mutant fibroblasts (<5% S-phase cells). Fig. 3 reports the pool sizes of the individual lines, with the dCTP pool representing in all instances the largest pool and dGTP representing the smallest. The size of each cytosolic dNTP pool varied among the different lines, but on average dGTP corresponded to 5.7 ± 0.3% (mean ± S.E.) of the total cytosolic dNTPs in WT fibroblasts and to 5.1 ± 0.5% in the mutants. The single recurrent difference between WT and mutant cells was a somewhat higher content of dTTP in the latter. We do not have at present an explanation for this difference (Fig. 3*A*). Quantitative variations of each dNTP were present also in the mt pools both within the controls and the mutant lines (Fig. 3*B*). The mutant cells contained less mt dGTP than the WT cells. The fraction of the mt pools corresponding to dGTP was 17.6 ± 2.4% in WT and 10.8 ± 1.4% in mutant cells. To evaluate how this difference reflected on the pool composition of each line, we normalized the individual pool sizes by that of the dGTP pool (Fig. 3, *C* and *D*). The composition of cytosolic pools did not differ statistically in mutant and WT fibroblasts (Fig. 3*C*), but this procedure revealed that the mitochondria of mutant cells contained an excess of dCTP and that dTTP and dATP tended to be more abundant relative to dGTP than in WT cells, although not significantly (Fig. 3*D*). Thus dGK deficiency, possibly changing the dynamics of the mt dGTP pool through a reduction of GdR phosphorylation (37), affected mt dNTP pool balance. The combination of lower dGTP concentration and mt pool imbalance might account for the mtDNA depletion. Much larger DNA precursor asymmetries observed in rat tissue mitochondria were shown to reduce the replication fidelity of human mt DNA polymerase when tested *in vitro* (32). Here, in cultured human fibroblasts, a milder mt pool imbalance was associated with mtDNA depletion.

**FIGURE 4.**
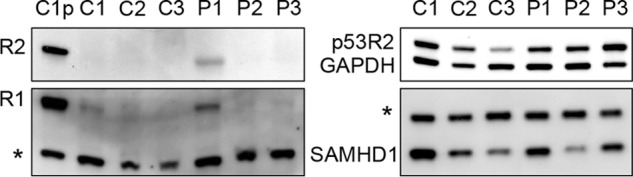
**Expression of ribonucleotide reductase subunits and SAMHD1 in untreated quiescent skin fibroblasts.** Ribonucleotide reductase subunits (*R2*, *p53R2*, and *R1*) and SAMHD1 were detected by immunoblotting in extracts from one proliferating WT control culture (*C1p*) and quiescent cultures of three WT (*C1*, *C2*, and *C3*) and three dGK-mutated (*P1*, *P2*, and *P3*) lines of skin fibroblasts. To compare R2 or R1 in proliferating and quiescent extracts, different amounts of proteins were loaded in the respective lanes of the gels: 4 and 40 μg for R2 and 20 and 40 μg for R1. In the case of p53R2 we used 2 μg of quiescent extracts and 20 μg for SAMHD1. Loading controls for R1, R2, and SAMHD1 were two unspecific bands (*asterisks*), whereas GAPDH was used as the control for p53R2.

We then compared dGK mutant and control fibroblasts for their expression of ribonucleotide reductase and SAMHD1 during quiescence in the absence of any experimental treatment. In protein extracts from three lines of WT fibroblasts and the three dGK mutant lines, we measured by Western blotting the expression of the three subunits of ribonucleotide reductase and of SAMHD1 (Fig. 4). The RNR small subunit p53R2, which increases in quiescent cells, was expressed in all lines, whereas the R2 subunit, which is S-phase-specific, was undetectable in all quiescent extracts with the exception of the P1 sample that gave a faint signal. The large subunit R1 produced much weaker bands in extracts from quiescent than from proliferating cells, and again the P1 extracts contained more enzyme than the other lines. All lines expressed SAMHD1, with similar variability in WT and mutant cells. The lowest expression was detected in C3 and P2 fibroblasts. The P1 fibroblasts had medium-high levels of SAMHD1 and higher expression of RNR subunits R2 and R1 than the other two dGK-mutated lines, a combination that may explain their higher mtDNA content (Fig. 2). We decided to test if lowering SAMHD1 activity in the cells with lower ribonucleotide reduction could increase their mtDNA.

##### Silencing of SAMHD1 in dGK-mutated Fibroblasts Prevents mtDNA Depletion during Quiescence

**FIGURE 5.**
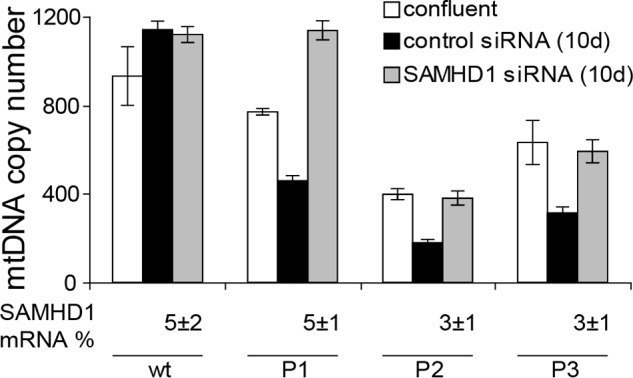
**Effect of SAMHD1 silencing on mtDNA content of quiescent skin fibroblasts.** mtDNA copy number normalized per nuclear genome was determined in WT and dGK-mutated fibroblasts at confluence, *i.e.* before starting the transfections with siRNAs in low serum medium and after 10 days of transfection during quiescence with control siRNA or anti-SAMHD1 siRNA. The values of three WT lines (*C1*, *C2*, and *C3*) were averaged, whereas those of the patient lines (*P1*, *P2*, *P3*) are reported separately. The percent residual level of SAMHD1 mRNA in the silenced cultures is indicated. Data are the means ± S.E. from three to six experiments for each cell line analyzed in triplicate.

Confluent cultures of WT and dGK-mutated fibroblasts were transfected with anti-SAMHD1 siRNA or control siRNA in medium with 0.1% serum and kept in the presence of siRNAs for 10 days, with 2 changes of medium. In a preliminary experiment we tested in one mutant (P1) and two WT lines three different anti-SAMHD1 siRNAs for the effects on SAMHD1 mRNA and mtDNA copy number. All three siRNAs silenced SAMHD1 to the same extent, with a residual mRNA level below 10% of the control (not shown). The amount of mtDNA remained the same in the WT fibroblasts when SAMHD1 was silenced (Table 2). On the contrary, in P1 fibroblasts mtDNA increased almost 3-fold to the level of the controls, suggesting that the down-regulation of dGTP catabolism by SAMHD1 prevented the depletion of mtDNA during quiescence. We also repeated the experiment with the other two mutant lines using siRNA 7 that in all instances decreased the level of SAMHD1 mRNA to ≤5% that of the control. Fig. 5 shows mtDNA copy numbers at the time of confluence, when siRNA transfections started, and after 10 days of transfection with control or anti-SAMHD1 siRNAs. In dGK-mutated fibroblasts transfected with control siRNA, the depletion of mtDNA, already present at confluence, became more marked after 10 days of quiescence. In contrast, SAMHD1 down-regulation led to a recovery of mtDNA that was complete in the case of P1 cells and only partial in the other two lines.

**TABLE 2:** **mtDNA copy number in quiescent control and dGK-mutated skin fibroblasts transfected with one nontargeting siRNA or three different anti-SAMHD1 siRNAs** mtDNA content was measured by quantitative real-time PCR and normalized per nuclear genome. Values are the means of three technical replicates for each condition ± S.E. from six experiments for control siRNA and siRNA 7 and from two experiments for siRNA 3 and siRNA 4. C1 and C2, controls; P1, dGK-mutant fibroblasts. ND, not done.

Cell line	mtDNA copy number			
	Control siRNA	siRNA 3	siRNA 4	siRNA 7
C1	1178 ± 73	1484 ± 80	1140 ± 74	1097 ± 67
C2	1100 ± 32	1548 ± 26	nd	991 ± 31
P1	461 ± 22	1372 ± 11	1390 ± 75	1070 ± 49

Earlier we showed that the expansion of the dNTP pools that results from silencing of SAMHD1 in quiescent fibroblasts depends on the activity of p53R2-dependent ribonucleotide reductase (10). Here the level of p53R2 was similar in the three mutant lines; thus, the lower level of mtDNA in P2 and P3 fibroblasts may depend on the lower content of R1 compared with P1 fibroblasts that in addition retained traces of R2 (Fig. 4).

**FIGURE 6.**
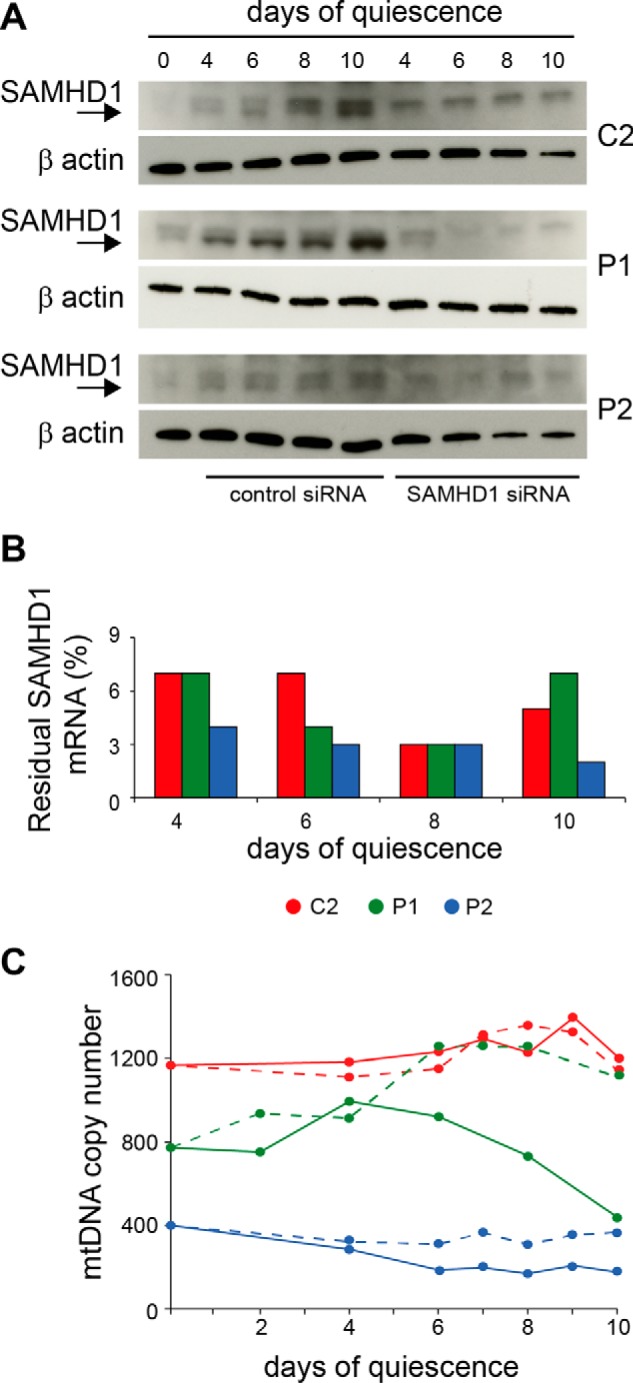
**Quantitative variations of mtDNA during siRNA silencing of SAMHD1 in quiescent WT and dGK-mutated fibroblasts.** *A*, expression of SAMHD1 protein during transfection with control or anti-SAMHD1 siRNA in quiescent cultures of WT (*C2*) or dGK-mutated (*P1*, *P2*) fibroblasts. The *arrow* indicates the SAMHD1 signal. An unspecific band appeared just above SAMHD1 and remained stable during silencing. *B*, residual level of SAMHD1 mRNA in the silenced cultures relative to that in the cultures transfected with control siRNA was determined at the indicated days of transfection during quiescence. *Red*, C2; *green*, P1; *blue*, P2. *C*, mtDNA content per nuclear genome was measured at 1- or 2-day intervals in cultures treated with control siRNA (*continuous line*) or anti-SAMHD1 siRNA (*broken line*) during 10 days of quiescence. Color coding is in *B*.

To understand how the boosting effect on mtDNA copy number developed during SAMHD1 silencing, we followed for 10 days of transfection with siRNA the variations of mtDNA in one line of WT fibroblasts and in the dGK-mutant fibroblasts with the maximum (P1) and minimum (P2) recovery of mtDNA. Transfections with control or anti-SAMHD1 siRNAs started at day 0 when the cultures had reached confluence and continued for 10 days during which we measured the levels of SAMHD1 protein (Fig. 6*A*) and mtDNA (Fig. 6*C*), every second day starting from day 4. Between days 4 and 10 of transfection we controlled the degree of silencing by quantitating SAMHD1 mRNA (Fig. 6*B*). In the cultures transfected with control siRNA, SAMHD1 increased with time as expected (10), more in WT and P1 cells than in P2 fibroblasts, which as observed above in Fig. 4 had the lowest endogenous expression of SAMHD1. In the cultures transfected with the anti-SAMHD1 siRNA, the protein became undetectable after day 4, reflecting the high degree of mRNA silencing (Fig. 6*B*). In agreement with the data in Table 2, the WT fibroblasts maintained their content of mtDNA constant during the whole period independently of the presence and concentration of SAMHD1. In the mutant fibroblasts the depletion was already present at day 0 and during transfection with nontargeting siRNA mtDNA decreased further, more markedly in P1 fibroblasts that started from a higher copy number. In P1 cells the down-regulation of SAMHD1 lead to an actual increase of mtDNA, whereas in P2 fibroblasts it only prevented the additional decline of mtDNA, possibly because these cells originally contained less SAMHD1. A similar stabilization of mtDNA content was obtained previously in a different line of dGK-mutated fibroblasts incubated with purine deoxynucleoside monophosphates (51).

##### Silencing of SAMHD1 Corrects the Imbalance of mt dNTP Pools in dGK-mutated Fibroblasts

**FIGURE 7.**
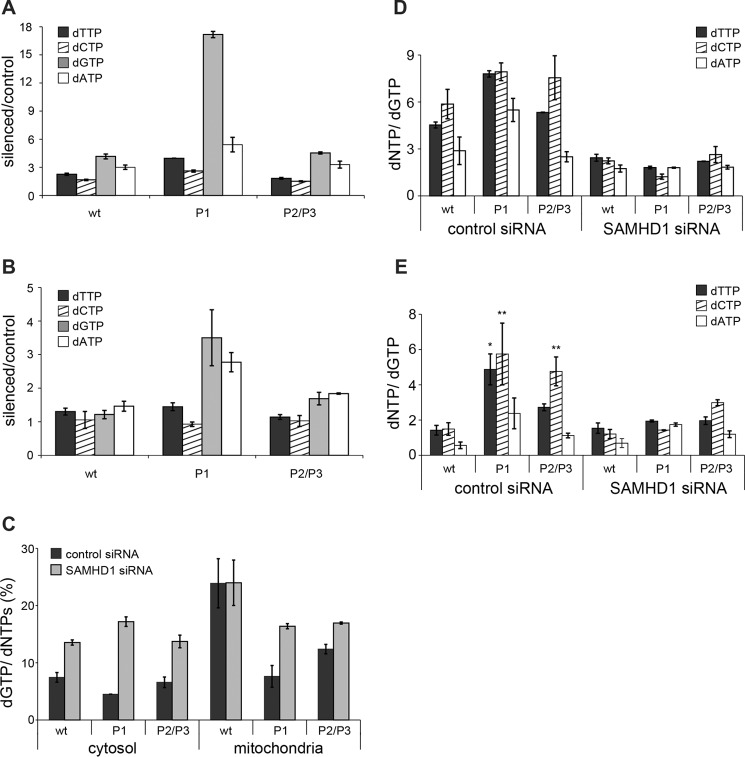
**Effects of SAMHD1 silencing on cytosolic and mitochondrial dNTP pools in quiescent WT and dGK-mutated fibroblasts.** Shown is the increase of cytosolic (*A*) and mitochondrial (*B*) dNTP pools induced by SAMHD1 silencing in WT and dGK-mutated skin fibroblasts incubated during 10 days of quiescence with anti-SAMHD1 siRNA or control siRNA. The increases measured in three WT lines were averaged as well as those of the P2 and P3 mutant lines. Values of the P1 mutant are reported separately. *C*, percentage of dGTP relative to the total dNTPs in the cytosolic and mt pools in WT and dGK-mutated fibroblasts transfected with control siRNA (*black*) or with anti-SAMHD1 siRNA (gray). *D* and *E*, composition of cytosolic (*D*) and mitochondrial (*E*) dNTP pools in cultures transfected with control siRNA or with anti-SAMHD1 siRNA, expressed as ratios between the sizes of the individual dNTP pools and the corresponding dGTP pool. The ratios of WT cells were averaged. Mutant P1 values are reported separately from those of the other two mutants (P2/P3). Data are the means ± S.E. from two experiments for each cell line analyzed in duplicate. *Asterisks* indicate that in nonsilenced cultures significant differences exist in the dTTP/dGTP and dCTP/dGTP ratios of dGK-mutated fibroblasts (P1, P2/P3) relative to controls (wt). **, *p* < 0.01; *, *p* < 0.05.

The improvement of mtDNA copy number in the mutant fibroblasts silenced for SAMHD1 suggests that their mt dNTP pools might be normalized as a consequence of reduced extramitochondrial catabolism of dNTPs. However, we previously found that SAMHD1 down-regulation in WT quiescent fibroblasts leads to larger and imbalanced dNTP pools (10), and Fig. 3 shows that the mt pools of dGK-mutated fibroblasts are imbalanced. Thus it was important to assess the effects of SAMHD1 down-regulation on these pools. In WT fibroblasts SAMHD1 silencing induced the expected changes in the cytosolic pools, with a larger expansion of purine dNTPs, a doubling of dTTP content, and only a minor increase of dCTP (Fig. 7*A*). mt pools were minimally affected (Fig. 7*B*). The average percentage of dGTP relative to the total dNTPs increased in WT cells from 7.4 ± 0.9 to 13.5 ± 0.5 in the cytosol, remaining unchanged in the mitochondria (24 ± 4) (Fig. 7*C*). The three mutant lines reacted differently to SAMHD1 silencing. Whereas the pool changes of P2 and P3 fibroblasts were close to those of the WT cells both in the cytosol and in mitochondria (Fig. 7, *A* and *B*), P1 cells expanded their purine dNTP pools to a much larger extent, in particular the cytosolic dGTP pool (Fig. 7*A*). The higher expression of ribonucleotide reductase in these cells during quiescence (Fig. 4) led to larger pools and complete recovery of mtDNA content (Figs. 5 and 6). Nevertheless, in all three mutant lines SAMHD1 down-regulation increased the percentage of dGTP relative to the total dNTPs not only in the cytosol, as in WT cells, but also in mitochondria (Fig. 7*C*). Despite the different quantitative alterations, in the mutant lines the composition of mt pools was normalized by SAMHD1 silencing, becoming similar to that of the WT cells. We report in Fig. 7, *D* and *E*, the cytosolic and mt pool composition relative to the content of dGTP for each cell line transfected with nontargeting or anti-SAMHD1 siRNA, calculated as in Fig. 3. In the cytosol the increase of dGTP content reduced the disequilibrium with the other three dNTPs similarly in WT and mutant fibroblasts (Fig. 7*D*). In mitochondria the large difference between mutant and WT cells disappeared, with a clear normalization of the pools in mutant cells (Fig. 7*E*). The correction of mt pool imbalance coincided with the observed improvement of mtDNA copy number.

##### Inhibition of Purine Nucleoside Phosphorylase Does Not Change mtDNA Content in dGK-mutated or WT Fibroblasts

The GdR derived from dGTP hydrolysis by SAMHD1 can be further degraded by PNP and thus subtracted from recycling via dGK-mediated salvage (Fig. 1). We performed experiments with quiescent WT and dGK-mutated fibroblasts in which during the last 24 h of transfection with control or anti-SAMHD1 siRNA we added to the medium 0.5 μm immucillin H, a potent chemical inhibitor of PNP (47). The prediction was that if PNP and dGK compete for their common substrate GdR, PNP inactivation may favor GdR incorporation into the mt dGTP pool of dGK-proficient cells but not of the dGK mutants. However, the treatment with immucillin H produced only minimal increases of cytosolic dGTP in the WT fibrobasts and had no effect on mt dGTP independently of SAMHD1 knockdown (data not shown). These negative results indicate that in cultured fibroblasts PNP interferes negligibly with salvage of endogenous GdR by dGK, and its inactivation does not modify the loss of dGTP due to SAMHD1 activity.

## Discussion

Genetic deficiency for either of the two mt deoxynucleoside kinases, TK2 and dGK, is associated with mtDNA depletion and severe mt diseases (41). The phenotypes differ for the two enzymes; TK2 mutations primarily affect skeletal muscle, and dGK mutations affect liver and brain. As the genetic defects are in each case present in all somatic cells, their specific phenotypic expression is ascribed either to specific energetic requests of the affected tissues that would require fully functional mitochondria or to variable expressions of the affected protein and other competing or cooperating enzymes. One example is the particularly low expression of TK2 in skeletal muscle, which makes this tissue specifically sensitive to any further decline of TK2 activity (52, 53), but in most cases the reason of the tissue-specific phenotype remains hypothetical. The expression of dGK, for instance, is comparable in liver and brain and in other tissues, which suggested that the specificity of the dGK^−^ phenotype might depend on differential expression of other enzymes functionally interacting with dGK (42). TK2 and dGK deficiencies affect non-dividing cells where *de novo* synthesis of dNTPs is strongly down-regulated and depends on the R1-p53R2 form of ribonucleotide reductase. In cultured human fibroblasts p53R2-dependent ribonucleotide reduction occurs at a roughly 40-fold lower rate than R2-dependent reduction during S-phase, yet the produced dNTPs largely exceed the needs of the cells during quiescence. In fact ≈90% of these dNTPs are dephosphorylated and the resulting deoxynucleosides are released into the medium (54). Thus in noncycling WT cells *de novo* synthesis of dNTPs *per se* does not appear to be insufficient for the maintenance of mtDNA. Nevertheless, the existence of the two mitochondrial deoxynucleoside kinases demonstrates that deoxynucleoside salvage is needed to support mtDNA synthesis under such conditions, but why are two distinct kinases required, and why inside mitochondria? At the end of S-phase TK1 is degraded, thus the need for a second thymidine kinase is obvious, although it may well reside outside mitochondria. In contrast to TK1, dCK is expressed with some variation in every phase of a cell's life and in all tissues, and it can phosphorylate both deoxyguanosine and deoxyadenosine besides deoxycytidine, its preferred substrate (3). Yet the cells also produce dGK, a purine-specific kinase enclosed in the mt matrix essential for the maintenance of mt dNTP pool balance during quiescence. In fact, differently from cultured TK2-deficient fibroblasts that do not manifest any mtDNA defect (55, 56), dGK-deficient fibroblasts readily develop imbalance of mt dNTPs (Fig. 3, see Ref. 39) and mtDNA depletion in quiescent cultures (Fig. 2*A*; Refs. 39 and 51). Why are noncycling cells so dependent on GdR salvage inside mitochondria? Our present data suggest that the answer may be SAMHD1, whose concentration increases in non-dividing cells (10). SAMHD1 is a nuclear enzyme, but its influence spreads to the overall cellular dNTP pool due to the permeability of the nuclear envelope to deoxynucleotides. Indeed, the anti-HIV-1 effect of SAMHD1 was attributed to the restriction of cytosolic dNTP pools that fall below the *k_m_* of the viral reverse transcriptase (7, 13). By degradation of all dNTPs in cells with low RNR activity, SAMHD1 restricts the source of deoxynucleotides for mtDNA synthesis. We demonstrated earlier that mt dNTPs largely derive from the cytosolic pool (37, 44). Two main dNTP transporters are known in mammalian cells, SLC25A33 and SLC25A36, both active with pyrimidine (d)NTPs, the latter also with (d)GTP (33). The deoxynucleosides produced by SAMHD1 enter mitochondria via nucleoside transporters (35, 36) and can be recycled by TK2 and dGK. In the mt matrix the resulting deoxynucleotides are sheltered from SAMHD1 and can be used for mtDNA replication or re-exported to the cytosol (34). Thus intramitochondrial salvage may be seen as a cell-autonomous mechanism (primarily) using deoxynucleosides derived from dNTPs synthesized *de novo* in the cytosol and hydrolyzed by SAMHD1 in the nucleus. By this means quiescent or differentiated cells would protect themselves from viral infections and at the same time secure balanced amounts of precursors for mtDNA maintenance. For the sake of the present argument we do not consider here the lower amounts of deoxynucleosides produced from nucleoside monophosphates by 5′-nucleotidases (4, 57). Phosphorylation of deoxynucleosides derived from the extracellular compartment might be a secondary function of mt deoxynucleoside kinases.

SAMHD1 is especially effective in restricting the size of the dGTP pool. When SAMHD1 is inactivated dGTP undergoes the highest relative increase compared with the other three dNTPs, implying that recycling of GdR is especially important to provide mitochondria with sufficient dGTP. The low concentration of dGTP in the cytosol of quiescent fibroblasts may reduce the rate of carrier-mediated import of dGTP into mitochondria so that recycling of GdR becomes limiting to sustain a “normal” mt pool.

With the present experiments we show that SAMHD1 contributes to the phenotype of dGK deficiency in fibroblasts. *In vivo* the main target cells of this genetic condition are hepatocytes and neurons, where the relative abundance of enzymes in the network outlined in Fig. 1 possibly differs from that in fibroblasts, our experimental model. The expression of cytosolic dCK, for instance, is particularly low in liver and brain (58, 59), increasing the dependence of the mt dGTP pool on intramitochondrial salvage of GdR. The tissue specificity of dGK deficiency *in vivo* may be related, at least in part, to SAMHD1 expression. When the gene coding for the protein now known as SAMHD1 was first described, it was reported to be highly expressed in liver on the basis of multi-organ Northern blotting analysis (60). However, a recent study of the protein expression profile in humans demonstrated that the intensity of the SAMHD1 signal in an organ mostly depends on the frequency of cells of hematopoietic origin, which express the highest level of SAMHD1, whereas liver cells contain little SAMHD1 (61). On the other hand, SAMHD1 is only one enzyme in the regulatory network, and its influence on the final concentration of dGTP depends also on the relative activities of other enzymes. The importance of ribonucleotide reductase in this respect is highlighted here by the biochemical phenotype of the P1 fibroblasts, which had higher RNR expression than the other two mutant lines (Fig. 4) and could better recover their mt pool balance and mtDNA upon SAMHD1 knockdown.

By silencing SAMHD1 during quiescence we managed to abolish or contain the mtDNA depletion caused by dysfunction of dGK. Previous studies have demonstrated the possibility of improving mtDNA copy number in cultured dGK-mutated cells by supplying high concentrations (≥50–100 μm) of purine deoxynucleosides (GdR ± deoxyadenosine) or their monophosphates in the medium of quiescent fibroblast cultures (Ref. 62 and references therein). The two protocols did not substantially differ, as nucleoside monophosphates are dephosphorylated by ectonucleotidases, and only the resulting deoxynucleosides are able to enter the cells. In dGK-deficient cells GdR (and deoxyadenosine) can only be phosphorylated in the cytosol by dCK. However, only at high concentrations did GdR compete with intracellular CdR for phosphorylation by dCK, whose *K_m_* for CdR is at least one order of magnitude lower than that for GdR (3). High concentrations of purine deoxynucleosides are also needed in these experiments to saturate the catabolic activity of adenosine deaminase and purine nucleoside phosphorylase. As an alternative, the two enzymes can be blocked by specific inhibitors. Here we tried to favor recycling of endogenous GdR produced by SAMHD1 by treating cells with immucillin H, but we did not observe any reproducible change in dGTP concentration. Our conclusion is that in cultured skin fibroblasts the enzyme does not have a measurable influence on the regulation of endogenous dGTP and that the degradation of exogenous GdR observed earlier by us and others (37, 62) is carried out mostly by the PNP present in the culture medium. With the present experiments we have updated the picture of the regulation of mt dGTP in human fibroblasts revealing a functional interaction between SAMHD1 and dGK that changes our perspective on the role of mitochondrial salvage of deoxynucleosides.

## Author Contributions

V. B. and C. R. conceived and coordinated the study and wrote the paper. E. F. and C. R. designed and performed the experiments. E. F. analyzed all the experiments and prepared the figures. C. S. contributed to the cytofluorometric analysis. E. F., V. B., and C. R. critically revised the manuscript for important intellectual content. All authors analyzed the results and approved the final version of the manuscript.

## Footnotes

3 The abbreviations used are:

RNR: ribonucleotide reductase
SAMHD1: sterile α motif/histidine-aspartate domain-containing protein 1
dCK: deoxycytidine kinase
TK1: cytosolic thymidine kinase
TK2: mitochondrial thymidine kinase
dGK: deoxyguanosine kinase
PNP: purine nucleoside phosphorylase
CdR: deoxycytidine
GdR: deoxyguanosine
p53R2: p53-inducible R2 subunit of ribonucleotide reductase
R2: ribonucleotide reductase subunit 2
R1: ribonucleotide reductase subunit 1
mt: mitochondria.
